# Analysis of soil water movement inside a footslope and a depression in a karst catchment, Southwest China

**DOI:** 10.1038/s41598-017-02619-x

**Published:** 2017-05-31

**Authors:** Hongsong Chen, Ke Hu, Yunpeng Nie, Kelin Wang

**Affiliations:** 1 Key Laboratory of Agro-ecological Processes in Subtropical Region, Institute of Subtropical Agriculture, Chinese Academy of Sciences, Changsha, Hunan 410125 China; 2 Huanjiang Observation and Research Station for Karst Ecosystems, Chinese Academy of Sciences, Huanjiang, Guangxi 547100 China; 3 University of Chinese Academy of Sciences, Beijing, 100049 China; 4 Water Resource Office, Hunan Provincial Water Conservancy Bureau, No. 370, North Shaoshan Road, Changsha, 410007 China

## Abstract

Soil water movement is difficult to explain with event-scale approaches, especially in karst regions. This paper focuses on investigating seasonal recharge and mean residence time (MRT) of soil water based on temporal variation of stable isotopes (δD and δ^18^O) and a dispersion model (DM), and discussing their differences along a footslope and a depression in a small karst catchment of southwest China. Temporal variations of the stable isotopes in precipitation and soil water within 0–100 cm profiles were monitored weekly for approximately 43 and 99 weeks. Results show that the seasonal recharge of soil water inside the footslope and the depression were similar, but the vertical flow velocity was higher implying a faster hydrological process in the footslope. The MRT of soil water (2–64 weeks) increased roughly, suggesting decreasing velocity of water displacement with increasing depth. However, the MRT at 60–100 cm depths in the depression (47–64 weeks) was obviously longer than at other sites, revealing more intensive water mixing. Furthermore, a shallower isotopic damping depth was found in the depression, indicating stronger delay and attenuation effects on base flow recharge. These results provide new insights into research on hydrological processes in karst areas.

## Introduction

Water movement in unsaturated soil zones plays a complex and important role in the transformation of precipitation to groundwater^1–3^. In a karst environment, soils are thin and rocky, and solution-enlarged fissures, gaps, and channels in the underlying bedrock facilitate the rapid transport of surface water to groundwater^4–8^. The strong interaction between surface and subsurface waters in karst areas makes soil water movement more difficult to decipher than in non-karst areas^3, 9^. Soil water movement is influenced by multiple environmental factors, such as precipitation, evaporation, vegetation, topography, and soil properties^10–13^. However, in karst areas, the spatial distribution of soil and permeable underlying bedrock is more heterogeneous, resulting in more complex hydrological processes, and then the effects of topography and landform may be more important than in non-karst areas^3, 14, 15^. Therefore, a more complete understanding of soil water movement in karst regions will help us manage shallow groundwater resources and deepen our knowledge of water balance in karst catchments.

Hillslopes and depressions are fundamental landscape units. Depressions are lower than hillslopes (a part of a hill between the top and the foot) surrounding it, and is identified as an even level place with deeper soil at the bottom of the catchment where a natural creek often appears. Depressions can be differentiated from upslope zones by their unique hydrology, vegetation, and soils^16, 17^. Water movement parameters such as flow path, mean residence time (MRT), recharge, and runoff generation have been frequently reported for hillslopes in many regions^18–20^. However, the water movement between hillslopes and depressions is particularly difficult to study in a karst environment. The high rock fragment content of soils and the heterogeneous underlying epikarst on the slopes make the soil water flow path complex and difficult to decipher^21–24^. High infiltration rates and the rare occurrence of overland flow on karst slopes indicate a rapid interaction between surface and subsurface water in the shallow soil zones^23, 25–27^. In contrast, soils in the lower parts of a hillslope (footslopes) and depressions are relatively thick and homogenously distributed. These characteristics imply that, compared to upslope regions, footslopes and depressions contain larger amounts of soil water, which can be investigated more easily. Moreover, hydrologic connectivity with stream networks exhibits seasonal variation on hillslopes, but not in the footslopes and depressions^9, 28^. Consequently, the differences in water movement in a footslope versus a depression will be more easily revealed in karst regions.

Traditional physical methods and approaches of hydrologic observation, like hydrograph and hydrochemistry methods, are insufficient for studying hydrologic patterns in heterogeneous karst regions^2, 4, 6^. Furthermore, traditional methods cannot be used to track water patterns under complex flow conditions in an unsaturated zone. However, stable isotopic methods are able to gather information about infiltration, evaporation, transpiration, and percolation under different soil conditions^2, 9, 29, 30^.

Stable isotopes, such as deuterium (D) and oxygen-18 (^18^O) have been powerful tools for researching the seasonal dynamics of soil water, stream flow, and spring flow at the catchment scale since the 1980s^31–34^. These methodologies are based upon distinct seasonal δD and δ^18^O patterns observed in input rainfall, which are roughly characterized by sine waves, and traced in output water^35–37^. Sine-wave functions, fitted to seasonal input and output isotope data, are used to determine the isotopic damping depths and MRTs of subsurface water sources^19, 35^. The seasonal variations of stable isotopes in precipitation are often attenuated in soil because of mixing and diffusional exchange with water that is stored in the soil pores. When the loss of seasonal variation is sufficient, the isotopic damping depth (the depth at which annual isotopic composition variations drop below 1/e or 37%) is defined, and identifies the flow length of precipitation that infiltrates, travels, stays still and disperses through the soil zones^19, 35^. Furthermore, this depth is useful for calculating the annual average hydraulic diffusivity, which triggers soil water movement, and for determining the intensity of horizontal flow^35, 38^. Obviously, topography and land formation would influence the damping depth and MRT, as well as other factors affecting soil water movement.

Based on seasonal variations of δD and δ^18^O, lumped mathematical residence time models can be used to evaluate MRT of soil water and time-based distributions^37, 39–41^. The dispersion model (DM) has been found to be the most appropriate means for calculating MRT in a soil zone at different depths, and DM can be approximated with fissured porous media^18, 41^. In order to get a better model fit, the DM also can reveal realistic distributions of MRT. McGuire *et al*.^42^ found that landscape organization parameters, such as flow path length and gradient, were more important for calculating catchment-scale MRTs than the contributing area. However, Soulsby *et al*.^32^ claimed that the influence of catchment topography was largely mediated by its influence on soil cover and distribution. Soil hydrology exhibits a strong relationship with MRT in catchments^43^. Thus, the influence of topography on soil water movement acts indirectly through catchment-scale hydrologic processes. In addition, the formation of less permeable soil, which could decrease the event recharge rate, tends to occur under lower slope gradients^44^, although seasonal recharge could be influenced by event recharge. Besides slope gradient, the contributing area, rock fragment content, and epikarst water (all of which are highly correlated with slope position) might also affect soil water movement in karst regions. However, few studies^9, 34^ have investigated the impact of slope position (e.g., footslope and depression) on seasonal recharge, damping depth, and MRT of water in soil zones.

The overall objectives of this paper are to evaluate water movement and storage in unsaturated soil zones by means of seasonal recharge and MRT of soil water in the footslope and depression of a small karst catchment in northwest Guangxi in southwest China. The results of this seasonal stable isotope hydrology study were used to address the following questions: (1) What is the relationship between the footslope and depression? (2) How do seasonal recharge and MRT differ between the footslope and the depression? and (3) Do topographic factors exert control over seasonal recharge and MRT of soil water?

## Results

### Seasonal variations in precipitation and isotopic relationship between rainfall and soil water

**Figure 1:**
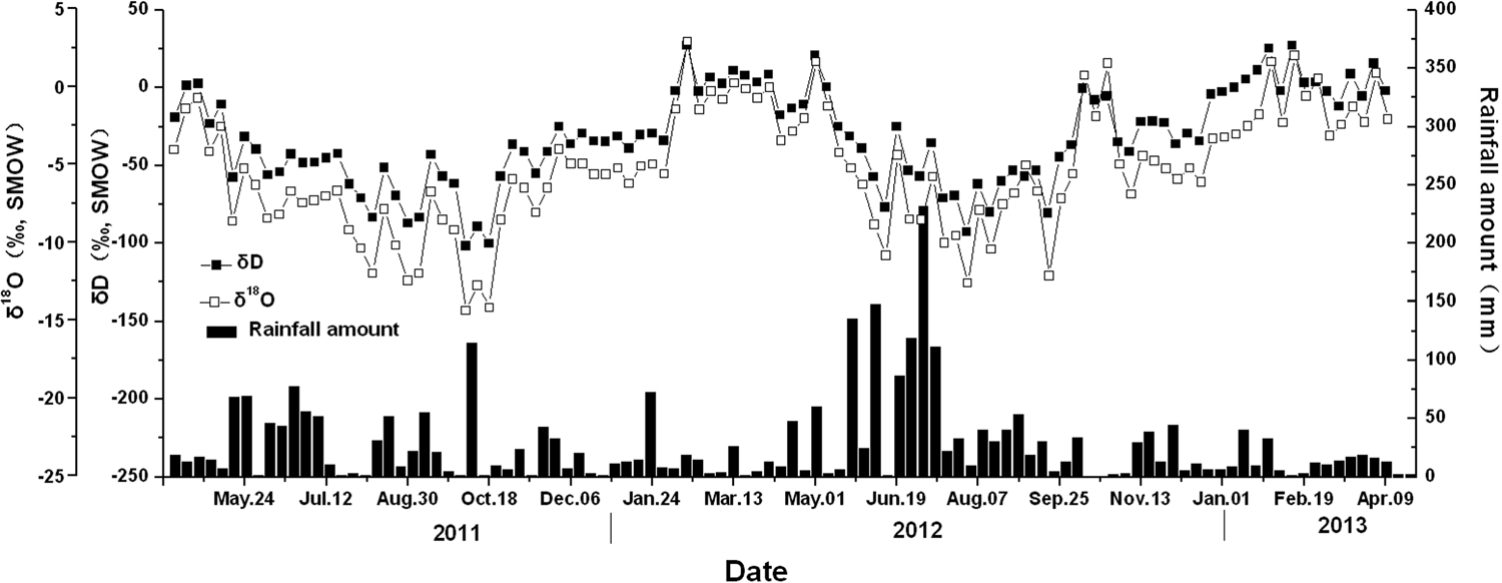
Precipitation amount and deuterium and oxygen-18 contents in rainfall with weighted weekly means from April 10, 2011 to February 9, 2013.

Rainfall amount and isotopic compositions of rainwater (δ^18^O and δD) displayed obvious seasonal variations from April 10, 2011 to February 28, 2013, as shown in Fig. 1. Rainfall mainly concentrated in rainy season (from April to September) with an average weekly value of 39.5 mm, which contributed 71.8% of the total precipitation during the 2-year sampling period. However, the dry season (from October to March) had an average weekly rainfall amount of 16.0 mm, and its maximum was less than 100 mm. The levels of *δ*D and *δ* ^18^O in weekly rainwater exhibited strong seasonal variations and, when graphed, presented the characteristics of sinusoidal waves (two sinusoidal cycles with 128‰ and 16‰ peak-to-peak variations, respectively) during the sampling period (Fig. 1). There was an inverse pattern between rainfall amount and isotopic composition, indicating high rainfall amounts corresponded to low *δ*D and *δ* ^18^O values. Hu *et al*.^45^ showed that smaller amounts of stable isotopes were found in heavy rainfall, but a one-year periodicity was observed and no linear relationship with event precipitation amount was exhibited. The calculation of seasonal recharge and MRT of soil water using input and output stable isotope data is based on the distinct stable isotopic signals of rainfall during the rainy and dry seasons.

**Figure 2:**
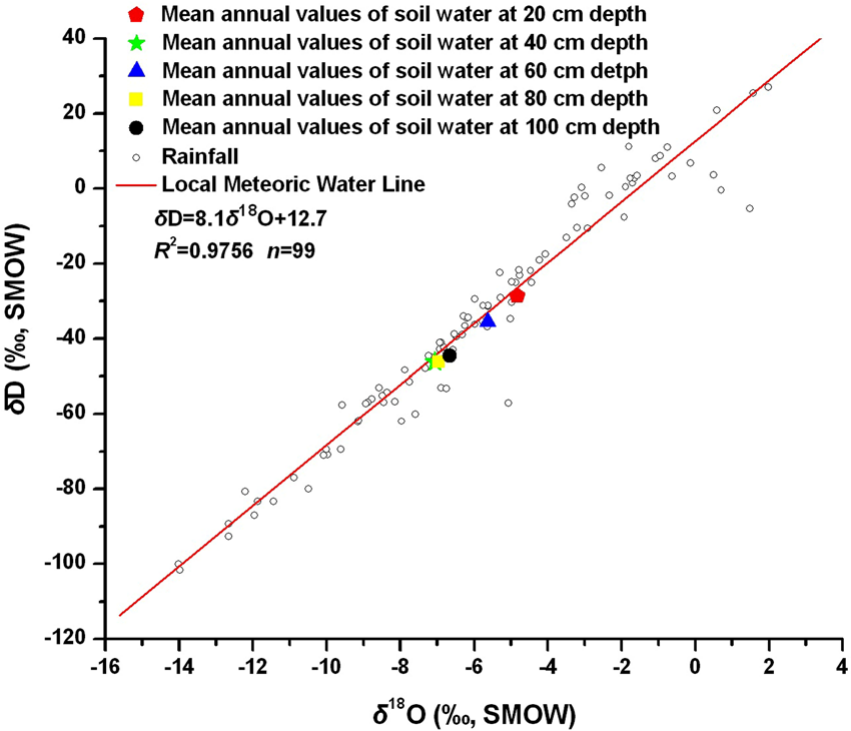
Relationship between δ^18^O and δD values of rainfall and soil water at depths of 20, 40, 60, 80, and 100 cm at the six sampling sites.

The local meteoric water line (δD = 8.1δ^18^O + 12.7; n = 99, R^2 = ^0.9756) was obtained from the weekly amounts of δ^18^O and δD values in rainwater between April 2011 and February 2013 (Fig. 2). The local meteoric water line was very similar to the global meteoric water line (δD = 8δ^18^O + 10). The mean annual δ^18^O and δD values of soil water at different depths were similar to both the local and global meteoric water lines, indicating that the non-equilibrium fractionation processes caused by evaporation could be negligible in the study area. Generally, evaporation of soil water was greater near the surface than in the deep soil layers because of surface heating^46^. However, the short interaction time between water and surface heating, caused by a rapid infiltration rate and less immobile water stored in shallow soil, seemed to lead to a below average evaporation rate. This allowed us to avoid overestimation of soil water recharge during the periods of high isotopic levels in input rainfall.

### Seasonal variation of stable isotopes in soil water at different depths

**Figure 3:**
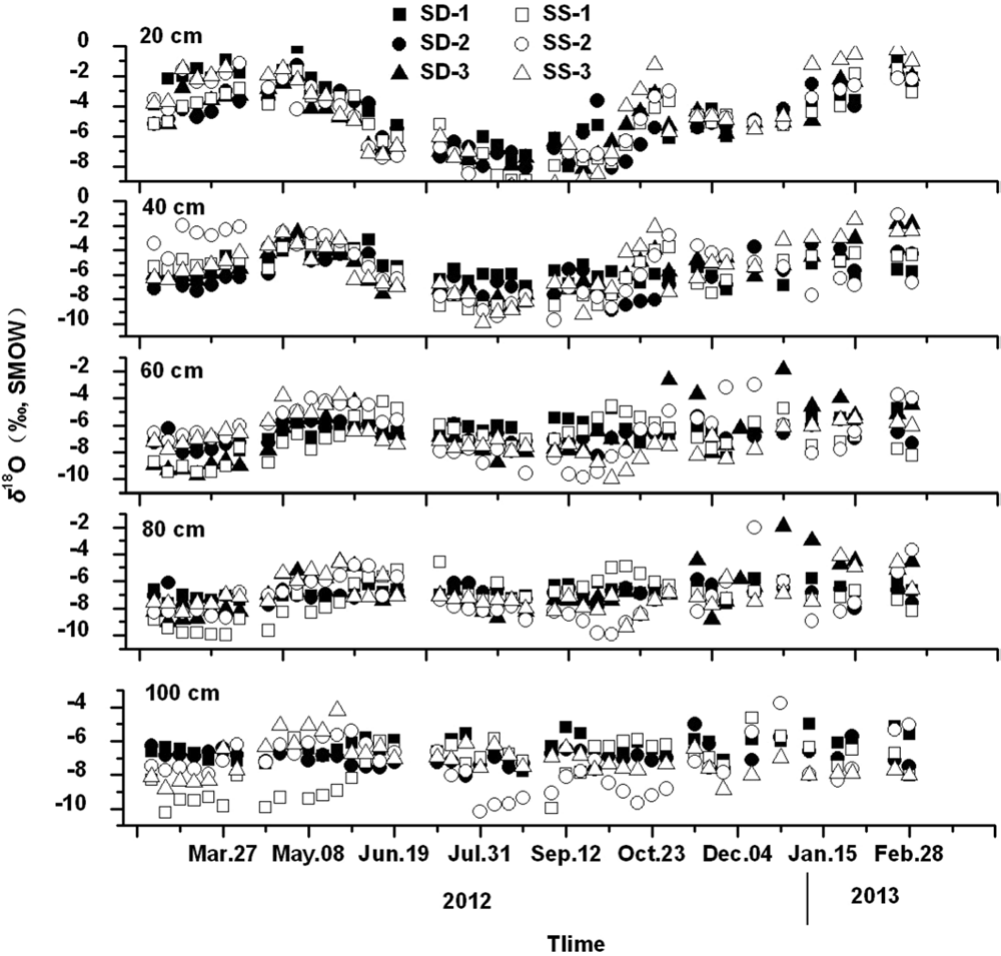
Seasonal variation of δ^18^O in soil water from February 21, 2012 to February 28, 2013.

**Figure 4:**
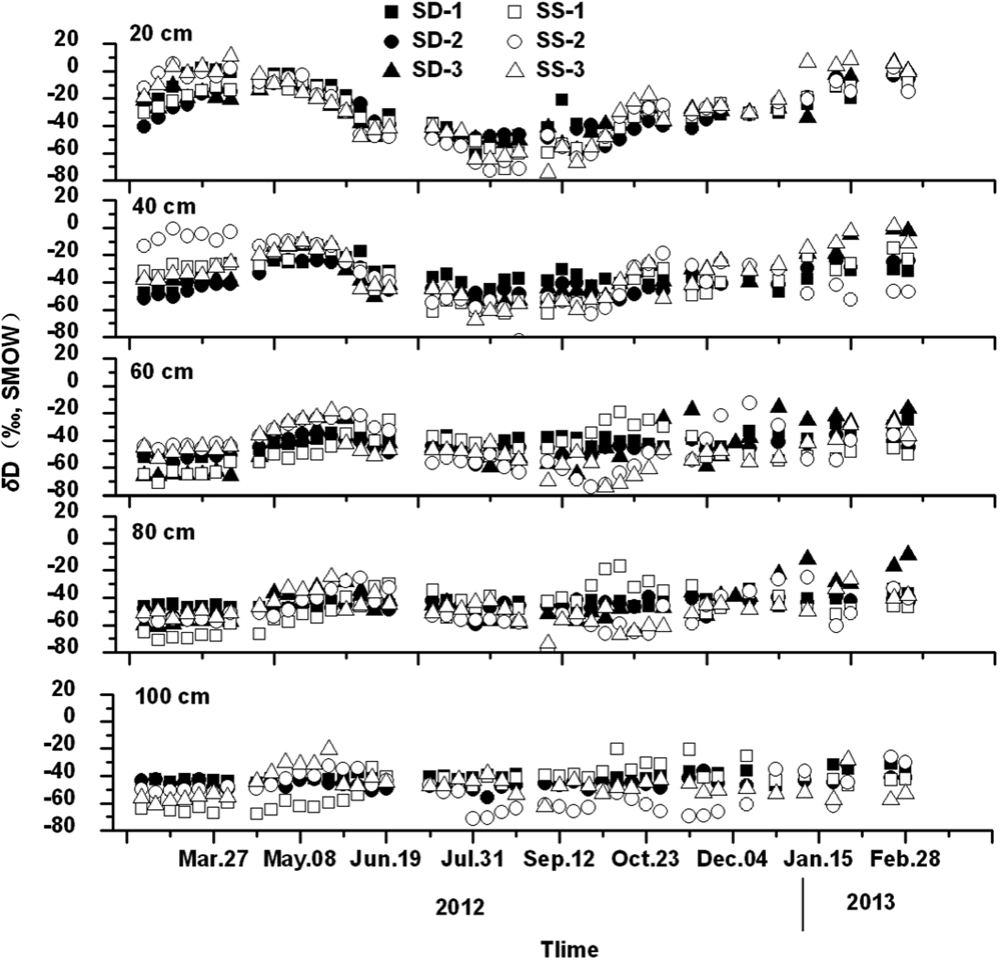
Seasonal variation of δD in soil water from February 21, 2012 to February 28, 2013.

Seasonal variations of *δ*D and *δ* ^18^O values for all soil water samples are given in Figs 3 and 4, respectively. As a whole, *δ*D and *δ* ^18^O values of soil water at each depth in both SD and SS sites were similar to each other, but differences existed among different soil depths. Variations of *δ*D and *δ* ^18^O values of soil water tended to decrease with increasing soil depth. At a depth of 20 cm, the *δ*D and *δ* ^18^O values of soil water expressed a similar sinusoidal wave like that of rainwater. At a depth of 40–60 cm, the *δ*D and *δ* ^18^O values of soil water showed a similar variation trend with that at a depth of 20 cm, but with a smaller varying extent. However, soil water at a depth of 80–100 cm showed steady-state *δ*D and *δ* ^18^O levels, with very little variation around the trend, indicating that a higher proportion of older water was present.

### Seasonal recharge of soil water

Mean annual *δ* ^18^O and *δ*D values of rainwater and soil water at different depths in all six sampling sites are shown in Table 1. The mean *δ* ^18^O and *δ*D values and their coefficient of variation (CV) of rainfall during the dry season were more enriched and had higher variation than during the rainy season. Generally, due to much larger rainfall amount during the rainy season, and therefore, the rainy-season rainfall should be the main recharge source for soil water compared to the dry-season rainfall. However, mean annual *δ* ^18^O and *δ*D values of soil water at a depth of 20 cm for all six sampling sites were close to those of annual rainfall, indicating an almost year-round recharge. Meanwhile, the highest CV at a depth of 20 cm reflected a significant response to rainfall. Therefore, this depth of soil could not contain much rainy-season rainfall or bypass flow. Soil water samples collected at a depth of 60 cm, showed depleted and lower *δ* ^18^O, *δ*D and CV values, respectively. The mean annual *δ* ^18^O and *δ*D values of soil water at 60–100 cm depths were near (or more depleted than) rainy-season rainfall values, illustrating that dry-season rainwater contributed little to recharge. However, there wasn’t a regular measurable difference between depression and footslopes. Soil water at a depth of 40–100 cm in SD-1 and SD-2 had lower CV than in the other sites. However, because of the presence of weathered sandy soil (Table 3), SD-3 was an exception in that it broke the above-mentioned tendency and exhibited the most significant response to rainfall than any other site at the same depth. In addition, compared with the footslopes, the mean annual *δ* ^18^O and *δ*D values of deep soil water (60–100 cm) in the depression were closer to those of baseflow. The annual *δ* ^18^O and *δ*D values of deep soil water in the footslopes were more depleted than those in baseflow.

**Table 1:** Summary of stable isotopes and CV in rainfall, baseflow, and soil water.

Samples	Depth (cm)	*δ* ^18^O (‰, SMOW)				*δ*D (‰, SMOW)			
		Min values	Max values	Mean values	CV	Min values	Max values	Mean values	CV
Rainfall		−14.01	1.98	−5.5	0.67	−101.56	27.17	−31.65	0.97
Rainfall_dry_		−14.01	1.98	−2.66	0.87	−101.56	27.17	−5.18	3.44
Rainfall_rainy_		−12.66	0.59	−6.38	0.59	−92.64	20.86	−43.93	0.64
Baseflow		−7.89	−4.99	−6.62	0.08	−33.92	−57.75	−42.34	0.12
SD-1	20	−8.01	−0.08	−4.36	0.49	−61.01	2.51	−25.82	0.69
	40	−6.96	−3.01	−5.56	0.18	−50.48	−17.03	−34.87	0.24
	60	−8.41	−4.43	−6.32	0.13	−52.34	−24.94	−40.98	0.16
	80	−7.52	−5.80	−6.75	0.07	−48.58	−33.45	−43.89	0.08
	100	−7.78	−4.97	−6.36	0.10	−47.72	−31.06	−40.56	0.09
SD-2	20	−8.12	−1.24	−4.96	0.38	−54.66	−2.90	−30.05	0.52
	40	−8.89	−3.16	−6.08	0.24	−54.89	−23.13	−40.07	0.25
	60	−8.26	−5.31	−6.75	0.10	−56.32	−35.30	−45.31	0.12
	80	−8.00	−5.86	−6.93	0.08	−59.55	−38.94	−46.03	0.09
	100	−8.07	−5.02	−6.92	0.08	−50.27	−31.29	−45.23	0.09
SD-3	20	−8.17	−1.14	−4.98	0.38	−57.68	6.95	−29.55	0.55
	40	−8.53	−1.81	−5.55	0.31	−54.55	−0.75	−33.56	0.43
	60	−9.67	−1.87	−6.65	0.28	−66.00	−15.92	−44.31	0.35
	80	−8.98	−1.91	−6.63	0.25	−60.98	−8.29	−43.24	0.32
SS-1	20	−8.98	−1.50	−5.07	0.41	−71.52	−0.64	−30.79	0.60
	40	−8.81	−3.44	−5.85	0.30	−62.55	−11.15	−37.25	0.41
	60	−9.49	−4.23	−6.92	0.20	−71.49	−19.24	−45.98	0.27
	80	−9.96	−4.59	−7.22	0.20	−71.03	−16.98	−46.56	0.29
	100	−10.25	−4.61	−7.49	0.19	−67.90	−20.68	−46.79	0.28
SS-2	20	−10.20	−1.14	−5.09	0.51	−72.93	2.57	−29.33	0.79
	40	−9.67	−1.10	−5.36	0.44	−82.96	−0.63	−33.78	0.61
	60	−9.85	−2.96	−6.47	0.28	−74.09	−12.73	−44.59	0.34
	80	−9.95	−2.04	−7.29	0.22	−78.82	−25.15	−49.81	0.25
	100	−10.02	−3.77	−7.44	0.19	−71.47	−25.86	−51.61	0.24
SS-3	20	−10.80	−0.24	−4.60	0.66	−74.32	8.97	−26.59	0.91
	40	−9.85	−1.49	−5.45	0.39	−61.34	1.24	−34.17	0.51
	60	−9.95	−3.82	−6.93	0.19	−74.32	−18.18	−46.48	0.27
	80	−9.38	−4.10	−6.96	0.17	−73.54	−23.71	−47.76	0.21
	100	−8.81	−5.09	−7.12	0.14	−62.65	−20.50	−46.99	0.19

Rainfall_dry_ and Rainfall_rainy_ indicate rainfall samples that were collected during dry and rainy seasons, respectively.

### Isotopic damping depth of soils

**Figure 5:**
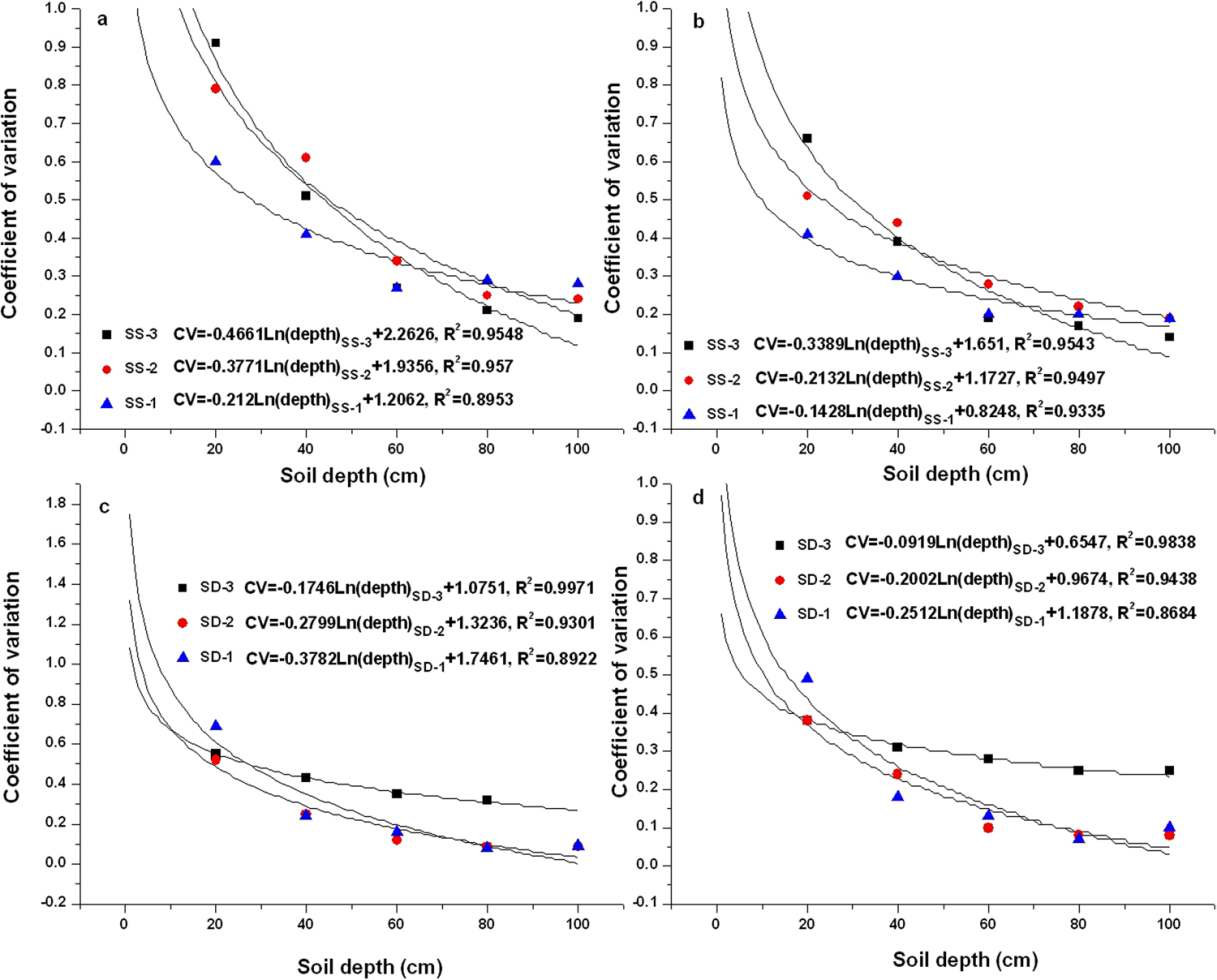
Logarithmic relationship between the coefficient of variation of annual deuterium (**a,c**) and oxygen-18 (**b,d**) contents and soil depth in each site from February 21, 2012 to February 28, 2013.

The annual CV of isotopic data for soil water decreased with increasing depth at each sampling site with a logarithmic relationship between the data and depth (Fig. 5). The isotopic damping depths obtained from *δ* ^18^O and *δ*D values show little variation, and thus, only *δ*D data were evaluated. The sharp decrease in CV at a 20 cm depth occurred in the depression but did not appear in the footslope. This suggests that most of the damping of the annual isotopic composition signal of precipitation occurred in the upper 0–20 cm soil layer in the depression. Based on the logarithmic relationship at each sampling site, the total isotopic damping depth can be calculated when the mean annual baseflow CV (0.12) is used^19, 35^. They were 73.6 cm in SD-1, 73.7 cm in SD-2 and 99.2 cm in SS-3, which were much shallower than those of SS-1 (167.9 cm) and SS-2 (123.3 cm). However, an abnormally high value (237.5 cm) was found in SD-3.

### MRT of soil water

To obtain MRT of soil water, seasonal variations of *δ* ^18^O and *δ*D in precipitation and soil water at different depths were used for DM simulations as input and output, respectively (Table 2). The infiltration rate, which reflected the difference of infiltration between rainy and dry seasons, was obtained and used to calculate MRT in soil water. The results calculated with *δ* ^18^O and *δ*D values had a few differences. Model efficiency (ME) of the DM based on *δ*D values was higher than that based on *δ* ^18^O values under most conditions, indicating a better fit of *δ*D than *δ* ^18^O. The MRTs of soil water ranged between 2 and 64 weeks and tended to increase with increasing depth. In the same sampling line, MRTs of soil water in the depression were longer than those in the footslope at depths of 40–100 cm. The sharp increases in MRT between depths of 40 and 60 cm took place in the depression, although they disappeared in the footslope. The MRTs of soil water at a 60 cm depth were more than three times longer than those at a 40 cm depth. However, there was very little difference between values for SD-1 and SD-2 at a 60−100 cm depth. SIGMA of simulations in the footslope was higher than those in the depression, indicating that the DM had a poorer fit in the footslope. ME functions for soil water at a depth of 60–100 cm were approximately equal to zero, indicating no periodic variation of the isotopes. MRT of soil water in SD-3, between 9 to 11 weeks (roughly increasing with depth), had no clear pattern. Moreover, SIGMA of simulations in SD-3 was obviously higher than that in SD-1 and SD-2, indicating a poorer fit. The estimated vertical flow velocity in soils was calculated from the ratio of soil depth to MRT. The 40–60 cm soil layer seemed to be a boundary zone, where water flowing through would move more slowly. However, opposite tendencies were observed in the SD-3 and SS-3 because of the presence of sand. In addition, the vertical flow velocities at a 100 cm depth in each sampling site displayed obvious variations: SD-1 and SD-2 had much lower values than SS-1, SS-2, and SS-3. The vertical flow velocities in the footslopes were 1.8–5.0 times faster than those in the depression. In addition, an opposite negative relationship between damping depth and MRT was found. There was a greater difference between MRT of soil water at the surface and deep layers, and a shallower damping depth was found in the surface layer.

**Table 2:** Parameters of fit DM models for oxygen-18 and deuterium variations in each sampling site from February 21, 2012 to February 28, 2013.

Site	Depth (cm)	*δ* ^18^O				*δ*D				FV (cm/week)
		T (weeks)	P_D_	ME	SIGMA	T (weeks)	P_D_	ME	SIGMA	
SD-1	20	4	0.55	0.7623	0.1894	6	1.20	0.8123	1.3770	3.33
	40	14	0.35	0.4655	0.1511	18	0.50	0.4353	1.2942	2.22
	60	47	0.27	NA	0.1172	57	0.12	NA	1.0327	1.05
	80	57	0.11	NA	0.0888	58	0.08	NA	0.9067	1.37
	100	64	0.14	NA	0.0923	60	0.11	NA	0.6451	1.56
SD-2	20	6	0.35	0.6933	0.1881	9	0.85	0.8253	1.1232	2.22
	40	9	0.05	0.3980	0.3033	12	0.60	0.3700	2.1837	3.33
	60	64	0.08	NA	0.1267	61	0.06	NA	1.3244	0.98
	80	58	0.10	NA	0.1250	57	0.08	NA	1.2732	1.40
	100	59	0.07	NA	0.1031	57	0.06	NA	0.9953	1.75
SD-3	20	8	2.40	0.8451	0.1300	10	2.50	0.8305	1.1958	2.00
	40	10	2.00	0.5759	0.2019	11	2.50	0.5672	1.9297	3.64
	60	10	0.01	0.0392	0.4611	10	0.01	0.1123	4.3131	6
	80	11	0.01	0.1398	0.4279	9	0.01	0.1649	3.9500	8.88
SS-1	20	6	0.85	0.8678	0.1205	10	2.00	0.9146	0.8454	2.00
	40	7	1.20	0.6243	0.2142	10	1.50	0.6505	1.7860	4.00
	60	22	0.04	0.2737	0.3066	24	0.03	0.3498	2.7765	2.50
	80	27	0.01	0.3985	0.3581	28	0.01	0.4513	3.0080	2.86
	100	33	0.01	0.3199	0.3620	31	0.02	0.5305	2.3959	3.23
SS-2	20	4	0.60	0.8833	0.1322	4	1.55	0.9228	1.0005	5.00
	40	4	0.20	0.7172	0.2206	3	0.15	0.5408	2.8823	13.33
	60	8	0.01	0.4995	0.3280	8	0.01	0.4777	3.2256	7.5
	80	9	0.01	0.3351	0.4191	11	0.03	0.1812	4.0874	7.27
	100	9	0.01	0.3001	0.4415	15	0.01	0.0688	4.4858	6.67
SS-3	20	2	0.60	0.8512	0.1752	3	1.35	0.9142	1.1120	6.67
	40	5	2.50	0.6081	0.2475	9	2.40	0.6816	1.7678	4.44
	60	9	0.01	0.3796	0.3806	9	0.01	0.3652	3.5505	6.67
	80	9	0.01	0.2072	0.4325	9	0.04	0.2480	3.6722	8.89
	100	11	0.01	0.1669	0.4085	12	0.01	0.1167	4.1931	8.33

T, P_D_, ME, SIGMA and FV were MRT, parameter of dispersion, model efficiency, accuracy of fit simulation and vertical flow velocity calculated from the data of *δ*D, respectively.

NA means that the model fits the data not better than a horizontal line through the mean concentration observed.

## Discussion

### Water recharge in the soil zone

The suction lysimeter samples contained water collected from the soils, and, thus, the preferential flow, which was difficult for plant use, could be avoided. The seasonal recharge of soil water was mostly caused by water stored in pores that had a relatively slow flow velocity^35^, and a recharge pattern that varied with depth. The 40–60 cm soil depth seemed to be a transitional boundary. On one hand, the water recharge of dry-season rainfall in the upper layer (0–40 cm) had a faster water flow velocity in the rainy season than in the dry season. On the other hand, in the lower layer (60–100 cm) the dry-season recharge was negligible and water mixed well. Lee *et al*.^11^ also found an analogous boundary between the upper fine-grained soil and the lower coarse-grained soil in Jeju Island, Korea, where soil water probably flows slowly through micropores rather than rapidly through macropores in the unsaturated soil zone.

The surface soil layer (0–20 cm) received an almost year-round recharge, indicating that the effect of rainy-season rainfall on surface layer was not as important as it was on deep layers. This is because the shallow soil had low water-holding capacity, and a large portion of rainy-season rainfall led to preferential flow instead of being stored in the micropores. Meanwhile, small quantities of water were stored in the upper soil layer where a fast pathway occurred. This pathway was not present in the lower layer. Previous studies have shown that root channels and other biopores (or cracks and fissures) may provide fast pathways for water movement in sandy soil or upper shallow loam soil^47–50^. In addition, the high content of rock fragments (usually distributed in the shallow soil zone) changes the soil pore volume and structure, which modifies the size and distribution of pathways for water movement through the soil zone^23, 51^.

The major recharge, occurring during the rainy season, profited from the fact that, relative to the upper layer, the lower soil layer has the ability to contain more water with a slower flow velocity. Soil physical properties such as BD, SWC, and CWC influence water movement, because they are impacted by soil pore properties. BD and pore-size distribution were two of the most important soil physical properties affecting infiltration and many aspects of the soil-water-plant-atmosphere system. They are often used to predict soil water retention properties^52–54^. However, both high macropore and micropore volumes can lead to low BD, although they have opposite impacts on water movement in soil zones. Hence, the relationship between BD and recharge processes is usually complicated. We tended to believe that macropores dominated the pores in shallow soil layers because plant roots, cracks, fissures, and other natural soil pipes, which form macropores, are likely to exist in shallow soil layers^47^.

Slope position did not have a consistent effect on soil water recharge. Spatial variability in soil characteristics and vegetation distribution likely had a greater influence on soil water than did slope position^55^. Soil water recharge had no obvious differences among different sampling sites between footslopes and depressions, and therefore, the recharge patterns were similar. However, soil water seemed to be mixed more sufficiently in the depression, perhaps owing to the occurrence of lateral flow. In a transition process from the driest to wettest conditions, the hydrologic connection between footslope and depression has been shown to be continuous owing to lateral flow, which has weak responses to rainfall^28, 56^. The interaction between epikarst water and soil water was strong in the deep soil zones. The transit times for the lateral water movement through bedrock were significantly greater than those for vertical movement of water through the soil^57^.

The weathered sandy soil controlled any abnormal soil water movement. Unlike ordinary soil, this kind of soil tends to have poor water-holding capacity, which results in fast infiltration. A set of mean saturated hydraulic conductivity values was developed according to soil texture, where the hydraulic conductivity value for sand was 15, 91, and 350 times greater than that for loam, clay loam, and clay, respectively^58^.

### Horizontal flow in the soil zone

The annual average soil hydraulic diffusivity exhibited a positive relationship with isotopic damping depth for soil water movement based on an analogy with unsteady heat flow^35^. They ranged from 93.25 to 485.27 cm^2^/d and were larger in the footslope than in the depression without a weathered sandy layer. The hydraulic diffusivities seemed to reflect water movement when soils had lower moisture contents^35^. This implied that horizontal flow in the footslope would occur more easily than in the depression.

The damping depth can be influenced by several factors, such as vegetation, soil porosity, rock fragment content, and topographic position. O’Driscoll *et al*.^19^ argued that interception and water uptake by vegetation during the growing period removed most of the water from the shallow soil layer, leading to a reduction in isotopic variation. However, the impact of vegetation was not significant because the sampling sites with analogous vegetation types did not have similar damping depths. On the contrary, the sampling sites with analogous slope positions (footslope and depression) had similar damping depths. This indicated that slope position might have a dominant impact on damping depth. The vertical component of kinetic energy, which is greater on gentle slopes, probably caused quasi-stagnant water that would have been stored in micropores (i.e., with slow flow velocity) to flow fast in the footslope^59^. Additionally, the weathered bedrock layer acted as a preferential flow channel as a result of the fractures distributed heterogeneously inside the layer, which made lateral flow easy^60^. The depression accumulated this lateral water from upslope areas, which had steady isotopic compositions, reducing the CV of soil water in deep layers. Rock fragment content and the weathered sandy soil layer seemed to have an opposite effect with vegetation: they supplied fast channels, allowing isotopic signals of soil water to exhibit a strong response to rainfall.

### MRT of soil water

The DM was used to interpret variations in *δ*D and *δ* ^18^O because it can be applied effectively to all soil water samples. As in some published case studies, interpretation of the D samples yielded P_D_ (dispersion parameter) values as high as 2.5^18, 42^. These high values reflect the high inhomogeneity and broad width of transit time distributions in shallow soil layers. This results from that the high rock fragment content, root system, and soil porosity provided varying flow pathways. However, the lower P_D_ values less than 0.05 were unexpected, which indicated that the DM produced poor matches to the data in a weathered sandy soil layer. Consequently, because of the probable wide distribution of weathered sandy soil, an appropriate improvement of the DM was necessary for application in karst catchments.

Vertical flow-path length had a weak influence on MRT of soil water because MRT exhibited no obvious linear relationship with soil depth. This result contradicts the viewpoint that MRT of soil water depends on the length of vertical infiltration^57^ and reflects the complex flow path in karst soil zones. The dominant factors controlling MRT of soil water were likely soil porosity and pore-size distribution^53^. Soils contained mobile water that is characterized by short MRTs in the fissures or large pores, and stagnant or quasi-stagnant water that is characterized by long MRTs in the micropores^48, 61, 62^. Consequently, soil drainable porosity appears to be an important control on new water ratios of hillslope discharge for steep, wet hillslopes with thin soil cover^20^.

Slope position (footslope or depression) has an important impact on MRT of soil water, resulting from its effects on slope gradient, rock fragment content, and water-contributing area. Asano *et al*.^57^ found that MRTs of soil water and transient groundwater were mostly described by soil depth, whereas, perennial groundwater and stream water, which were strongly affected by water flow through bedrock, can be described by the upslope contributing area. McGuire *et al*.^42^ found that simple topographic factors (such as gradient) were strongly correlated with water transfer at the catchment scale, despite the relatively complex hydrological processes involved. The slope gradient improved soil permeability and caused a high recharge coefficient^44, 63^. This relationship meant that the temporal variations of isotopic compositions in weighted rainfall were higher in the footslope than in the depression. The shorter MRTs of soil water resulted from the large variation of isotope values in input rainfall. Although previous studies have shown that landscape organization is a first-order control on MRT^42, 64^, its effect was not significant in the soil zones of the study area. The MRT of water increased with increase in upslope contributing area^57^. Footslopes maintained water tables and were almost continuously connected with the stream network, even in the dry season^28^. This observation suggests that recharge of soil water in the depression from footslopes through horizontal flow was continuous^60^. Moreover, the horizontal recharge of deeper soil water in the depression probably came from epikarst water, which had longer MRTs^9^. In addition, Chen *et al*.^23^ found that the mean total volumetric rock fragment content tended to have a positive relationship with slope gradient on hillslopes. Rock fragments might supply fast flow pathways and thus decrease the MRT of soil water. Ponding and runoff flow were delayed in soils with a high cover of rock fragments^22^. This finding indicates that rock fragments facilitated water infiltration and actually caused fast-flowing water, which was characterized by short MRTs.

Weathered sandy soil, which is similar to sand with high hydraulic conductivity and a wide pore-size distribution facilitating water infiltration^53, 58^, seems to be the reason for short MRT. Obviously, this weathered sandy soil had less capillary water, lower saturated water content, and poorer water-holding capacity, suggesting that fast flow controlled water movement. Consequently, it was not difficult to conclude that the soil hydrological function can be divided into three cases according to analysis of MRT of soil water and vertical flow velocity: (1) Soil in the footslope and at 0–40 cm depths in the depression is characterized by moderate water-holding capacity and vertical flow velocity; (2) Soil at 60–100 cm depths in the depression is characterized by good water-holding capacity and slow vertical flow velocity; and (3) The weathered sandy soil layer has the poorest water-holding capacity and the fastest vertical flow velocity.

## Conclusions

Soil water movement and storage in footslopes and a depression was evaluated with respect to seasonal recharge, isotopic damping depth, and MRT using temporal variations of *δ*D and *δ* ^18^O and a dispersion model (DM). Year-round recharge was found in soil water at a depth of 0–20 cm, whereas soil water at a depth of 40–100 cm was recharged seasonally. Recharge was more likely to occur during the rainy season. Water flow velocities of shallow soil layers (0–40 cm) were faster than those of deep soil layers (40–100 cm). The DM provided a better fit in ordinary soil rather than in weathered sandy soil. The MRTs of soil water in the footslopes ranged from 2 to 31 weeks. However, a MRT longer than 1 year at a depth of 60–100 in the depression implied that this layer was a well-mixed zone or was recharged from epikarst water. Higher vertical flow velocities and deeper isotopic damping depths were obtained in the footslopes. This demonstrates that average annual hydraulic diffusivity for water movement in the footslopes was larger than in the depression. Slope position (footslope and depression) had a significant impact on MRT of soil water and damping depth, but it only affected seasonal recharge slightly. The weathered sandy soil layer had poorer water-holding capacity and shorter MRT than ordinary soil. This sandy layer was found underneath the ordinary soil layer and above the epikarst and often exhibits a strong response to rainfall. The above results indicated that soil water movement was complex and distinctive in karst areas with high heterogeneity, and the spatial distribution of soils and vegetation should be considered in future hydrological modeling at a catchment scale.

## Materials and Methods

### Site descriptions

**Figure 6:**
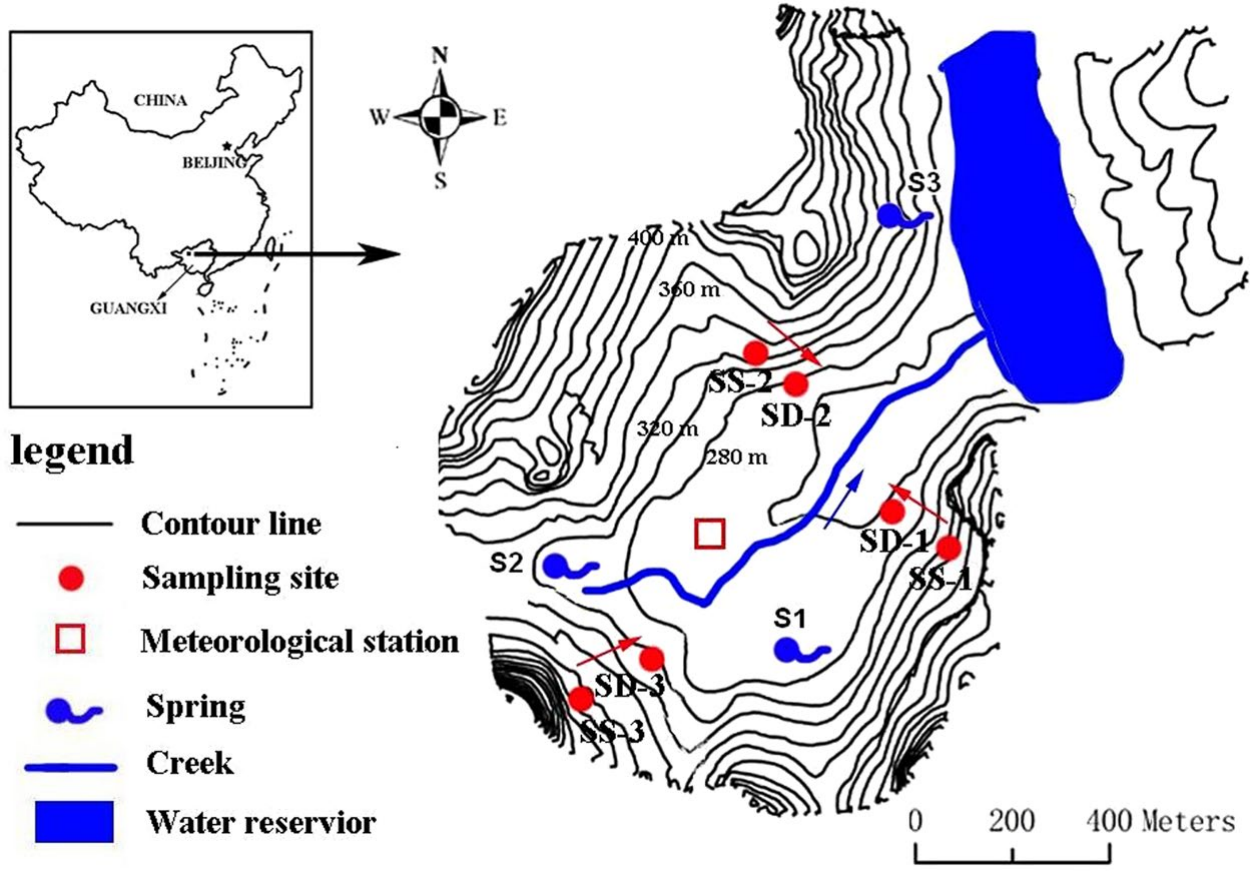
Schematic map of sampling sites in a small catchment with contour lines at 20-m intervals in Huanjiang County of Guangxi, China (modified from Fig. 3 in ref. 9). Schematic map of sampling sites was generated by software ArcGis 9.2 and ArcView 3.2, and the positions of sampling sites were located by GPS.

The study was conducted at the Huanjiang Observation and Research Station for Karst Ecosystems under the Chinese Academy of Sciences (24°43′–24°44′N, 108°18′–108°19′E) in Huanjiang County of northwest Guangxi, southwest China (Fig. 6). The experimental site is a typical peak-cluster depression area, which is characterized by a relatively flat depression with an elevation lower than 280 m above sea level (about 28% of the total catchment area) surrounded by overlapping hills and ridges except for an outlet in the northeast. It has a subtropical mountainous monsoon climate and covers an area of 1.01 km^2^. Approximately 60% of the slope gradients are larger than 25° and elevation ranges from 272 to 647 m above sea level. Mean annual temperature is 19 °C, and mean annual precipitation is 1389 mm, mostly occurring between May to early October. Soil depths in the depression and on the hillslope are 20–160 cm and 0–50 cm, respectively. On most of the hillslope tops, there is usually no soil cover and bedrock is exposed. The shallow and discontinuous soils have been developed from dolomite and contain significant amounts of rock fragments^23^. Soils are well drained, gravelly and calcareous, and have a clay to clay-loam texture (25–50% silt and 30–60% clay). Weathered sandy soil, underlying relatively impermeable rocks, sometimes appear in the deep soil layers both in the footslope and depression. Based on tension infiltrometer measurements (20 cm in diameter), stable infiltration rates range from 0.43 to 4.25 mm/min^26^. Organic matter content is relatively high ranging from 2.2% to 10.1%, and pH varies between 7.1 and 8.0. The average percent of exposed bedrock ranges from 15% in the depression to 30% on the hillslope. Some rock outcrops are large (2–10 m in height) with a vegetative cover of deep-rooted trees.

All residents have relocated and the cultivated lands have been abandoned since 1985. The dominant vegetation types are grass and sparse shrub. However, there are patches of zonal dense scrub and forest with a high amount of exposed bedrock, especially in the southwest. Overland flow on the hillslopes, under the various land cover types, is low and the corresponding runoff coefficient is often less than 5%^26^. Three seepage springs sometimes appear at the bottoms of the hillslopes in the rainy season and recharge the creek. The groundwater table changes seasonally and is often 1–3 m below ground surface in the depression^65^.

A creek originates from the southwest corner of the catchment where vegetation cover is relatively dense. This creek is linked with an excavated channel in the middle of the catchment. The outlet of the catchment is at the northeastern end of the channel. All of the surface water and part of the subsurface water flow into a small water reservoir in the northeast (Fig. 6).

### Rainfall and base flow sampling

Weekly rainfall samples were sequentially collected within the study area from April 10, 2011 to February 28, 2013. Precipitation sampling devices were adopted from IAEA and consisted of a 150-mm-diameter funnel, connected at the base to a 0.5 L brown bottle^9^. An air outlet tube was welded to the lower part of the funnel. To avoid evaporation of water samples, wax was used at every junction.

Water samples of creek base flow were collected at the outlet of the study catchment weekly from February 21, 2012 to February 28, 2013. A polyethylene bottle (2 mL) was used to collect the water samples and a loop was created in the bottle to prevent water vapor from migrating out of the sample reservoir.

### Soil water sampling

In order to investigate the effects of slope position, vegetation and soil properties on water movement, three sampling lines were placed along slopes with similar gradients (11–17°) as illustrated in Fig. 6. According to the landform and the distribution of soil depth and vegetation, each line had two sites chosen for soil water sampling, with one location in the footslope (SS) and the other in the depression (SD). SS-1, SS-2, and SS-3 were located in the footslopes, and the corresponding sites in the depression were SD-1, SD-2, and SD-3, respectively. Vegetation types consisted of woodland in SD-2 and SS-2 and shrubland in all other sites. The locations of the three sampling lines were selected to represent the vegetation types in the study area.

Soil water was sampled by use of tension lysimeters (produced by the Institute of Geographic Sciences and Nature Resources Research, Chinese Academy of Sciences), which consisted of suction lysimeters, ceramic porous cups, sampling bottles, tubes, and pressure monitors^66^. A small hand pump was connected to each device and an approximate 80-Kpa vacuum was created within the bottles. Soil water was collected at depths of 20, 40, 60, 80 and 100 cm using the ceramic porous cup and placed into the sampling bottles at each sampling site. In the SD-3, soil depth was less than 100 cm, thus no water sample was collected at a depth of 100 cm, and water samples at 60 and 80 cm depths were collected within the weathered sandy soil layer. Soil water was extracted at weekly intervals for 43 weeks from February 21, 2012 to February 28, 2013. At the same time, shallow groundwater levels were monitored in the depression at intervals of five and ten days during rainy and dry seasons, respectively. The groundwater table varied seasonally (i.e., shallow during the rainy season and deep during the dry season)^65^. The subsurface depth ranged from 0.18 m to greater than 5 m. This indicates that the distribution of precipitation strongly influences the groundwater table. In addition, a groundwater level of less than 1 m is only found from May to July under heavy rainfall. This indicates that most soil water samples were collected from unsaturated zones.

Soil physical properties, such as saturated water content (SWC), capillary moisture content (CMC) and bulk density (BD) were measured in 0–100 cm soil profiles to evaluate their corresponding impact on soil water movement and storage at the six sites along the three lines. Near each soil water sample site, a 1.2 m deep soil profile was dug (about 1 m deep in the SD-3). Due to the relative homogeneity of soil horizontal distribution, the two undisturbed soil samples were collected with an ordinary ring knife (100 cm^3^) at depths of 0–10, 10–20, 20–30, 30–40, 40–50, 50–70 and 70–100 cm, respectively. BD, SWC and CMC were measured in the laboratory for each undisturbed sample^9^. According to the vertical distribution of soil properties in the study area^15, 67^, the soil profile could be divided into three soil layers, 0–10, 10–50 and 50–70 cm for SD-3 and 0–10, 10–40 and 40–100 cm for other sites (Table 3).

**Table 3:** Soil physical properties within the soil profiles at the six sampling sites in the experimental area.

Site	Depth (cm)	SWC (%)	CMC (%)	BD (g/cm^3^)	Site	Depth (cm)	SWC (%)	CMC (%)	BD (g/cm^3^)
SS-1	0–10	68.37	53.13	0.97	SD-1	0–10	69.89	57.77	1.01
	10–40	48.74	45.42	1.03		10–40	55.08	52.22	1.20
	40–100	52.46	48.60	1.17		40–100	62.74	57.37	1.09
SS-2	0–10	57.56	49.04	1.05	SD-2	0–10	68.61	52.04	0.96
	10–40	49.31	42.10	1.05		10–40	50.38	45.68	1.18
	40–100	52.85	47.72	1.09		40–100	57.21	50.94	1.13
SS-3	0–10	64.77	49.73	1.00	SD-3	0–10	70.30	61.83	0.82
	10–40	49.05	45.05	1.28		10–50	68.10	56.90	0.83
	40–100	52.23	46.32	1.22		50–70*	18.68	14.47	1.39

The asterisk (*) indicates a weathered sandy soil layer. The others were ordinary soil layers, including loam and clay loam.

### Stable isotopes analysis

Deuterium and oxygen-18 values of the water samples were analyzed by the DLT-100 (Los Gatos Research (LGR), Inc., model 908-0008), a liquid water isotope analyzer, at the Key Laboratory of the Agro-ecological Processes in Subtropical Regions, Institute of Subtropical Agriculture, Chinese Academy of Science. Results were reported in δ notation relative to V-SMOW as:

1 $${{\rm{\delta }}}_{{\rm{sample}}}=({{\rm{R}}}_{{\rm{sample}}}/{{\rm{R}}}_{{\rm{standard}}}-1)\times 1000$$

where, R_sample_ and R_standard_ are the ratio D/H and ^18^O/^16^O of the measured sample and standard sample, respectively. The standard deviation for repeated measurements was ±1‰ for *δ*D and ±0.2‰ for *δ* ^18^O.

### Damping depth estimation

Damping and lagging of the seasonal fluctuations, with increasing depth or flow path length, in the subsurface soil and rock were used to compute an “isotopic damping depth” analogous to the damping depth computed for soil temperature fluctuations based upon sine-wave analysis^35^. Isotopic damping depth is defined as the soil depth at which the input signal needs to become damped to equal levels similar to the mean base flow at the catchment^19^. A functional relationship between input and output can be represented as^35^:

2 $${\rm{amplitude}}\,{\rm{change}}\,({{\rm{d}}}_{{\rm{h}}})={[\mathrm{ln}({{\rm{A}}}_{Z2}-{{\rm{A}}}_{Z1})/(Z2-Z1)]}^{-1}$$

where, d_h_ (damping depth) is in cm, A_Z2_ is the amplitude at depth Z2 in ‰, and A_Z1_ is the amplitude at depth Z1 = 0 in ‰.

### Mean water residence time analysis

To provide an understanding of the MRT of soil water at different depths and positions, a lumped parameter mathematical model was used: the dispersion model (DM), which is based on long-term isotope data. The DM was adopted because it has a better fit than other models when applied to a porous medium and an unsaturated soil zone^39^. The Flow PC software version 3.1, which was introduced from IAEA, was used^68–70^. A functional relationship between input and output can be represented as:

3 $${{\rm{C}}}_{{\rm{out}}}({\rm{t}})={\rm{\Sigma }}{{\rm{C}}}_{{\rm{in}}}({\rm{t}}^{\prime} ){\rm{g}}({\rm{T}})\exp (-{\rm{\lambda }}{\rm{T}}){\rm{d}}{\rm{t}}^{\prime} $$

Where, C_out_ and C_in_ are *δ*D and *δ* ^18^O values of soil water and rainfall samples, respectively; g(T) is the system response function, which specifies residence time distribution of water within the system; t′ is time of entry; and T = t–t′ is the MRT of water, which can be calculated from the system response function through the calibration of the models. Based on sufficient input data for C_out_ and C_in_, the g(T) can be calculated.

In flux mode of the DM, the following uni-dimensional solution to the dispersion equation for a semi-infinite medium is used as the response function

4 $${\rm{g}}({\rm{t}})={(4{{\rm{\pi }}{\rm{P}}}_{{\rm{D}}}{\rm{t}}/{\rm{T}})}^{-1/2}{{\rm{t}}}^{-1}\exp [-{(1-{\rm{t}}/{\rm{T}})}^{2}({\rm{T}}/4{{\rm{P}}}_{{\rm{D}}}{\rm{t}})]$$

where P_D_ is the apparent dispersion parameter, which mainly depends on the distribution of travel times. The higher values reflect more inhomogeneity and a greater breadth of transit time distributions. Consequently, T can be obtained by determining the best fit between output and input data.

The infiltration rate, which represents the fraction of precipitation entering the groundwater system in the observed month, is needed before the MRT is calculated with the Flow PC software. The accuracy of fit of simulations to the experimental data is computed using the SIGMA function:

5 $${\rm{SIGMA}}={\rm{\Sigma }}{({{\rm{S}}}_{{\rm{i}}}-{{\rm{M}}}_{{\rm{i}}})}^{2}/{\rm{n}}$$

where, S_i_ and M_i_ are simulated isotope values and measured isotope values, respectively; and n is the number of samples. It is obvious that the lower SIGMA value means a higher quality of fit for the simulations and suggests that the models are the most applicable.

The model efficiency (ME) is introduced in the version 3.1 of Flow PC according to Hornberger *et al*. (1992):

6 $${\rm{ME}}=1-{\rm{\Sigma }}{({{\rm{S}}}_{{\rm{i}}}-{{\rm{M}}}_{{\rm{i}}})}^{2}/{\rm{\Sigma }}{({{\rm{C}}}_{{\rm{mean}}}-{{\rm{M}}}_{{\rm{i}}})}^{2}$$

where, C_mean_ is the arithmetic mean of the measured values. Equation (6) is useful for testing breakthrough curves in artificial tracer tests and periodic output functions. In the case of stable isotopes, seasonal variations are used to find a model.
